# Innate Lymphoid Cells in Graft‐Versus‐Host Disease

**DOI:** 10.1111/ajt.13394

**Published:** 2015-07-30

**Authors:** V. Konya, J. Mjösberg

**Affiliations:** ^1^ Center for Infectious Medicine, Department of Medicine HuddingeKarolinska InstitutetStockholmSweden; ^2^ Institute of Experimental and Clinical PharmacologyMedical University of GrazGrazAustria

**Keywords:** Basic (laboratory) research/science, bone marrow/hematopoietic stem cell transplantation, graft‐versus‐host disease (GVHD), immunobiology, intestinal disease, lymphocyte biology, translational research/science

## Abstract

Innate lymphoid cells (ILC) are lymphocytes lacking rearranged antigen receptors such as those expressed by T and B cells. ILC are important effector and regulatory cells of the innate immune system, controlling lymphoid organogenesis, tissue inflammation, and homeostasis. The family of ILC consists of cytotoxic NK cells and the more recently described noncytotoxic group 1, 2, and 3 ILC. The classification of noncytotoxic ILC—in many aspects—mirrors that of T helper cells, which is based on the expression of master transcription factors and signature cytokines specific for each subset. The IL‐22 producing RORγt^+^ ILC3 subset was recently found to be critical in the prevention of intestinal graft‐versus‐host disease (GVHD) following allogeneic hematopoietic cell transplantation (HCT) via strengthening the intestinal mucosal barrier. In this review, we summarize the current view of the immunological functions of human noncytotoxic ILC subsets and discuss the potentially beneficial features of IL‐22 producing ILC3 in improving allo‐HCT efficacy by attenuating susceptibility to GVHD. In addition, we explore the possibility of other ILC subsets playing a role in GVHD.

## Abstract 2

Group 3 innate lymphoid cells are potential regulators of host sensitivity to graft‐versus‐host disease.

**Abbreviations**
Ahr: aryl hydrocarbon receptor
AMP: antimicrobial peptide
GVHD: graft‐versus‐host disease
HCT: hematopoietic cell transplantation
IFN: interferon
ILC: innate lymphoid cells
ISC: intestinal stem cells
KLRG1: killer cell lectin‐like receptor subfamily G member 1
LTi: lymphoid tissue inducer
NCR: natural cytotoxicity receptor
TNF: tumor necrosis factor
TSLP: thymic stromal lymphopoietin

## Development and Classification of ILC Subsets

**Figure 1:**
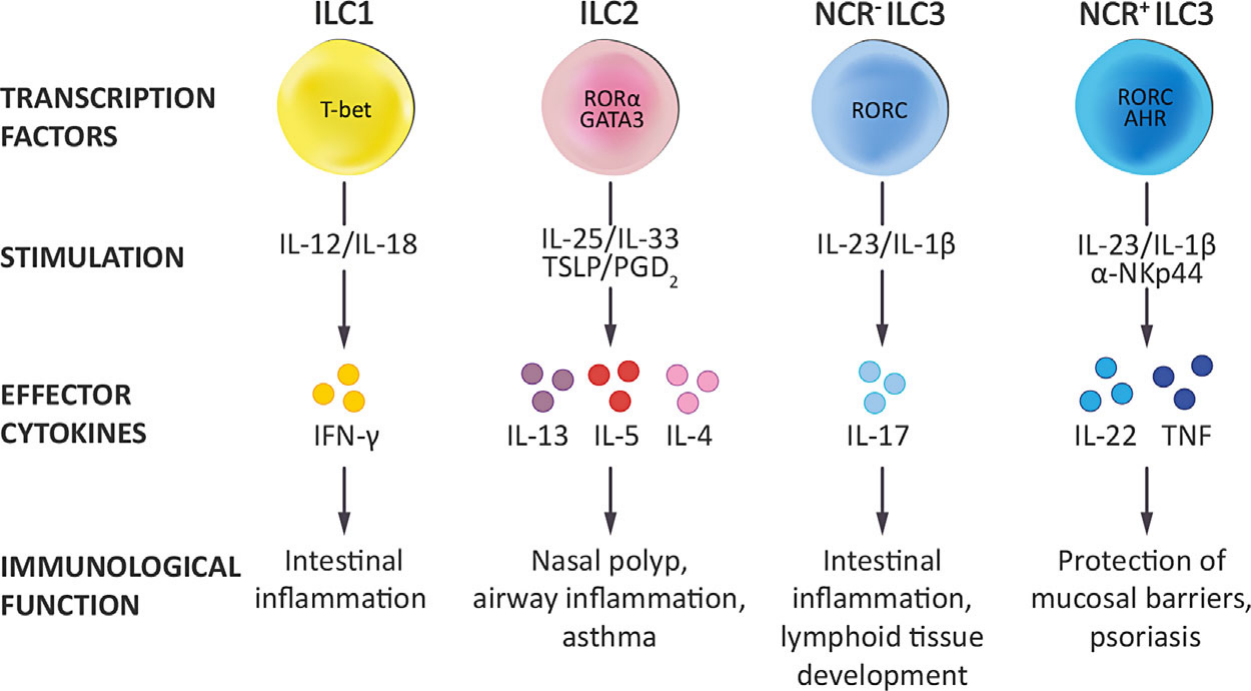
**The human ILC subsets** The classification of human innate lymphoid cells into group 1–3 ILC is based on their expression of specific transcription factors and cytokine production profiles. ILC, innate lymphoid cells; IFN, interferon; NCR, natural cytotoxicity receptor; TNF, tumor necrosis factor; TSLP, thymic stromal lymphopoietin.

Innate lymphoid cells (ILC) are effector cells contributing to the early immune responses against microbes 1, stimulating tissue regeneration 2 and strengthening the epithelial barrier at mucosal surfaces 3. A detailed description of the tissue‐specific functions of the human ILC subsets have recently been provided elsewhere 4. The family of ILC includes the cytotoxic natural killer (NK) cells, as known since the 1970s for their ability to kill target cancer cells and produce interferon (IFN)‐γ. In addition to NK cells, three major subsets of noncytotoxic ILC have been described during the last decade. The classification of these noncytotoxic ILC into group 1, 2, and 3 ILC is based on their expression of specific transcription factors and cytokine secretion profiles largely reflecting the Th1/Th2/Th17 subsets (Figure 1) 5. Additionally, ILC subsets are defined by the expression pattern of certain cell surface proteins, which are partly different in humans and in mice (Table 1).

**Table 1:** Cell surface phenotype of human and mouse ILC subsets

	CD127^−^ ILC		CD127^+^ ILC1		ILC2		NCR^−^ ILC3		NCR^+^ ILC3	
	human	mouse	human	mouse	human	mouse	human	mouse	human	mouse
CCR6	−	−	+	−	+/−	−		+/−	+	−
CD4	−	−	−	−	−	−	−	+/−	−	−
CD7	ND	ND	+	ND	+	ND	+	ND	+	ND
CD25	ND	ND	−	−	+	+	−	+	−	ND
CD56	+	NA	−	NA	−	−	−	NA	+/−	NA
CD90	ND	ND	ND	ND	ND	+	ND	+	ND	+
CD94	+	NA	−	NA	−	NA	−	−	−	+/−
CD103	+	−	−	−	−	−	ND	ND	−	ND
CD117	−	−	−	−	+/−	+	+	+	+	+
CD127	−	−	+	+	+	+	+	+	+	+
CD161	+	−	+	−	+	−	+	−	+	−
CRTH2	−	ND	−	ND	+	+	−	ND	−	ND
ICOS	−	ND	−	ND	+	+	ND	−	+	−
IL‐1bR	ND	ND	+	+	+	ND	+	+	+	+
IL‐12RB	ND	ND	+	+	−	−	−	−	−	−
IL‐23R	ND	ND	−	−	−	ND	+	+	+	+
NKp44	+	NA	−	NA	−	NA	−	NA	+	NA
NKp46	−	+	−	−	−	−	−	−	+	+
Sca1	NA	ND	NA	ND	NA	+	NA	+	NA	ND
ST2	−	−	−	−	+	+	−	−	−	−

NCR^+^ refers to NKp44^+^ ILC3 in human and NKp46^+^ ILC3 in mouse. Expression level of the cell surface markers is displayed as follows: + indicates high expression, − indicates no expression, +/− indicates bimodal expression. ND indicates that expression level is not defined and NA indicates that the marker is not applicable in the respective species.

ILC show lymphoid morphology but lack rearranged antigen‐specific receptors and lineage markers associated with other lymphocytes such as T and B cells. Alike these latter cell types, ILC develop from the common lymphoid progenitor (CLP). However, in contrast to T and B cells, ILC critically depend on the transcriptional repressor Id2 for commitment to the ILC lineage 6. The Id2‐dependent ILC progenitor (ILCp) or common innate lymphoid progenitor (CLIP) gives rise to all ILC subsets including NK cells. Furthermore, as a progenitor of ILC1, ILC2, and ILC3, the common helper‐like innate lymphoid progenitor (CHILP) was identified, which depends on the transcription factors PLZF, GATA3, NFIL‐3, and Notch 6, 7. The development of human ILC and their progenitor(s) are less well characterized. However, recently, the human progenitor of IL‐22 producing ILC3 was described as RORγt^+^ CD34^+^ hematopoietic stem cells selectively residing in the tonsil and intestinal lamina propria 8.

Recent observations associate one specific ILC subset, the IL‐22 producing ILC3, with reduced sensitivity to graft‐versus‐host disease (GVHD) after allo‐hematopoietic cell transplantation (HCT) 9, 10. Hence, this review provides an overview of the noncytotoxic ILC: ILC1, ILC2, and ILC3, with particular focus on the IL‐22 producing ILC3 as potential players in the prevention of GVHD.

## ILC Subsets

### ILC1

Group 1 ILC, similarly to NK and CD8^+^ T cells, depend on T‐bet and produce IFN‐γ and TNFα in response to IL‐12 and IL‐18 stimulation.

In humans, based on IL‐7Rα (CD127) expression, two ILC1 populations can be distinguished. The intraepithelial CD127^−^ ILC1, found in the human intestine, express CD56, CD94, NKp44, and CD103. This cytotoxic ILC1 subset exists in mouse as well, where it was found to express CD160 and NKp46 but not CD103. Furthermore, this subset was NFIL‐3‐, and T‐bet‐dependent and IL‐15‐responsive 11. Importantly, these cells, although cytotoxic, were shown to be distinct from NK cells as they also developed in *il15r^−/−^* mice lacking NK cells.

The second subgroup of ILC1 in humans, the CD127^+^ ILC1 was found in the inflamed human gut lamina propria, and do not express CD56, CD94, and NKp44 12. In the mouse, another CD127^+^ T‐bet^+^ NKp46^+^ Eomes^−^ non‐NK ILC1 lineage was identified 13. This particular ILC1 subset showed poor cytotoxicity, but was the major producer of IFN‐γ and TNF in response to Toxoplasma gondii intestinal infection.

ILC1 are most probably involved in inflammation by producing IFN‐γ upon sensing epithelial and myeloid cell‐derived danger signals. Both CD127^−^ and CD127^+^ ILC1 are highly accumulated in the inflamed ileum of Crohn's disease patients, potentially contributing to the pathogenesis of gut inflammation 11, 12.

### ILC2

ILC2 secrete type 2 cytokines; IL‐4, IL‐5, IL‐9, and IL‐13 in response to IL‐33, IL‐25, and thymic stromal lymphopoietin (TSLP) 14, 15, 16. Additionally, ILC2 produce amphiregulin, which is crucial in tissue regeneration 2. For the development and function of human and mouse ILC2, the transcription factors GATA3 and RORα are essential 17. In the peripheral blood, the majority of CD127^+^ ILC are ILC2, additionally, group 2 ILC have been found in the healthy human lung 2, 18 and in the skin 19, 20. An updated view on ILC2 function in pulmonary and dermal inflammatory diseases is provided elsewhere 21. In humans, ILC2 are abundant in nasal polyps of patients with chronic rhinosinusitis (CRS) and in bronchoalveolar lavage (BAL) of idiopathic pulmonary fibrosis patients 18, 22. In the skin, increased presence of ILC2 is associated with atopic dermatitis both in human and mouse as reviewed elsewhere 21. Furthermore, ILC2 were identified in the mouse adipose tissue regulating fat metabolism 14.

### ILC3

Group 3 ILC are characterized by IL‐22 and IL‐17 secretion in response to IL‐23 and IL‐1β stimulation 5, 23, 24. ILC3 express the transcription factor RORγt, and the development of mouse ILC3 depends on RORγt, Notch, and Tcf1, as recently summarized elsewhere 6. Additionally, the transcription factor aryl hydrocarbon receptor (Ahr) is crucial in the maintenance and function of ILC3 4. The first subset of ILC3 to be described was the lymphoid tissue inducer (LTi) cells, essential for proper lymph node formation during embryogenesis and for the formation of lymphoid follicles in the postnatal intestine, as extensively reviewed elsewhere 25. Human ILC3 can be further divided into two subsets based on expression of the natural cytotoxicity receptor (NCR) NKp44. NCR^+^ ILC3 are cellular sources of the tissue protective cytokine IL‐22 4. Additionally, engagement of NKp44 triggers TNF release providing a pro‐inflammatory function for NCR^+^ ILC3 26. The NCR^−^ ILC3 secrete little IL‐22 but do produce IL‐17, similarly to the LTi‐like ILC3 in the mouse 25. In the mouse, the ILC3 populations are differentially defined. Murine α4β7^+^ progenitors give rise to CCR6^+^ T‐bet**^−^** ILC3, such as LTi cells, which heterogeneously express CD4. The α4β7^−^ ILC3 progenitors depend on AhR for their development into CCR6^−^ T‐bet^+^ ILC3, which may or may not express the NCR NKp46 5.

NCR^−^ ILC3 make up one of the most frequent ILC subsets in the human peripheral blood, while NCR^+^ ILC3 are virtually absent in the circulation during homeostasis 20. Importantly, the healthy human intestine hosts dominantly NCR^+^ ILC3, which have been shown to be essential in maintaining gut homeostasis 1. The signature cytokine of NCR^+^ ILC3 is IL‐22, a cytokine belonging to the IL‐10 cytokine family. Exceptionally among cytokines, IL‐22 does not target immune cells, but stromal cells and epithelial cells at physical barriers, such as skin, gut, and liver 27. Mouse studies established a crucial role for IL‐22 in gut homeostasis, since ILC3‐derived IL‐22 protected against *Citrobacter rodentium*–induced colitis 1. Furthermore, IL‐22‐producing ILC3 in the gut were found to control anatomical containment of commensal bacteria, thereby protecting against intestinal inflammation 28. Intestinal epithelial cells express the IL‐22 receptor (IL‐22R) and IL‐22 directly induces production of the Reg family of antimicrobial proteins (AMPs) 29. Reg3β and Reg3γ are important regulators of epithelial barrier function and control the microbial colonization at epithelial surfaces. Further notions on how ILC3 and other ILC subsets may control intestinal homeostasis and inflammation are discussed elsewhere 27.

Interestingly in the skin, accumulation of IL‐22 producing NCR^+^ ILC3 is linked to dermal inflammation in psoriasis patients 21.

## Development of GVHD

The only curative treatment for hematologic malignancies nonresponding to induction chemotherapy is allogeneic HCT. However, alloreactive donor T cells can attack vital organs by recognizing recipient antigens, which leads to development of GVHD. The occurrence of GVHD is the major limiting factor in the therapy success of HCT and represents high morbidity and mortality. Prior to HCT, patients with malignant hematological disorders receive conditioning chemotherapy and radiotherapy in order to cytoreduce the malignancy and to deplete the nonmalignant host immune system to avoid rejection of the transplant. However, conditioning chemotherapy affects not only the immune cells but also the rapidly proliferating epithelial cells lining the mucosal barrier of the gastrointestinal tract and the skin 30. The consequence of compromised barrier function is uncontrolled spreading of commensal bacteria and tissue injury, which may further stimulate donor alloreactive T cells, and thereby aggravate tissue damage in the gut, skin, and liver 31. This syndrome is clinically recognized as acute (a)GVHD, which typically occurs within the first 3 months after transplantation. Major characteristics of aGVHD are activated antigen‐presenting cells (APC), pro‐inflammatory cytokine milieu, and enhanced recruitment and activation of effector T cells. Gastrointestinal aGVHD or mucositis is an especially important component of transplant morbidity and mortality, since it cannot be efficiently controlled by immunosuppressive corticosteroid therapy 32. Notably, the colonic mucosal tissue alterations of GVHD patients were reminiscent of chronic inflammatory bowel disease 33. The currently existing therapy of aGVHD focuses exclusively on the donor or graft immune responses against the recipient tissue 31 but the host responses during GVHD and the recipient‐specific potential therapeutic target mechanisms are very poorly understood. Additionally, the regulation of recipient GVHD sensitivity has not been addressed yet.

## Potential of IL‐22 Producing ILC3 in GVHD Therapy

### Lessons from mouse studies

**Figure 2:**
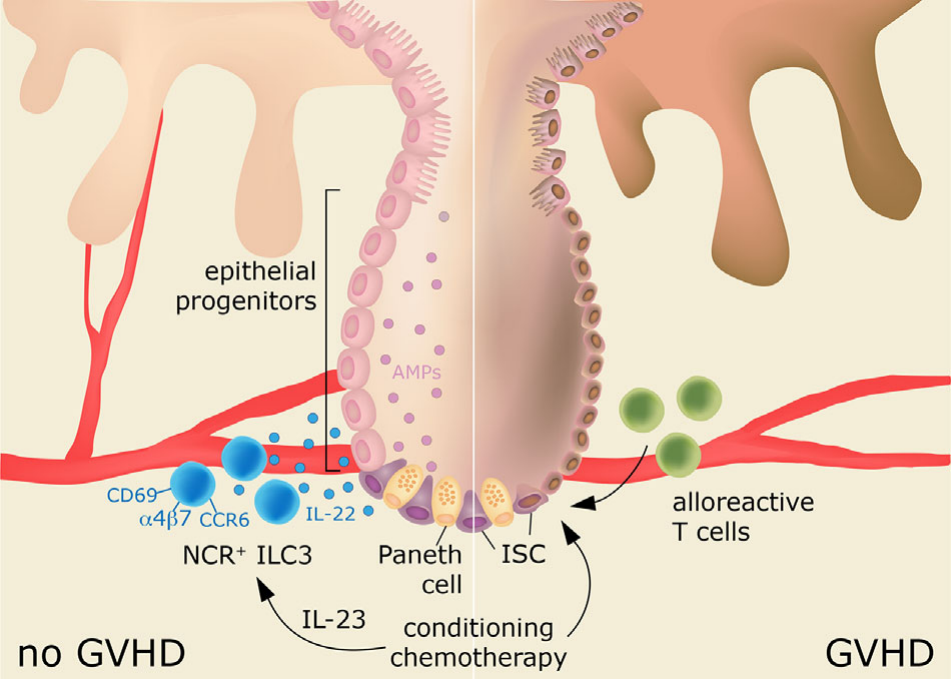
**NCR^+^ ILC3‐derived IL‐22 protects against GVHD after HCT** Conditioning chemotherapy causes damage to the intestinal crypt stem cells (ISC) and to the epithelial progenitors, which, via activation of alloreactive T cells, leads to intestinal GVHD (right panel). After HCT, NCR^+^ ILC3 expressing CD69 and the gut‐homing markers α4β7, CCR6 appear in the circulation most probably via upregulation of IL‐23. The activated NCR^+^ ILC3 produce IL‐22, which is required for the expression of antimicrobial peptides (AMPs) by the ISCs and the epithelial progenitors. AMPs limit the tissue damage caused by conditioning chemotherapy and immune depletion (left panel). Additionally, there are clues for an association between protection against GVHD and ILC2 and NCR^−^ ILC3 subsets, however, in a yet uncharacterized manner. NCR, natural cytotoxicity receptor; ILC, innate lymphoid cells; GVHD, graft‐versus‐host disease; HCT, hematopoietic cell transplantation.

The critical involvement of ILC in GVHD immunity was evidenced for the first time in a mouse model of acute intestinal GVHD, where IL‐22 producing ILC3 were found to attenuate GVHD 9. In particular, host IL‐22 deficiency resulted in increased incidence and severity of GVHD with excessive epithelial cell apoptosis and disrupted intestinal mucosal barrier. Furthermore, GVHD caused the most significant damage to the intestinal stem cells (ISC), which was even more pronounced in case of host IL‐22 deficiency. ISC are located at the base of the intestinal epithelial crypts between Paneth cells and ISC are able to generate the whole crypt‐villus structures *in vivo* and *in vitro* 34. The authors identified IL‐22R expression on ISC and epithelial progenitor cells, which—together with mature intestinal epithelial cells—produce antimicrobial proteins Reg3β and Reg3γ in response to IL‐22 29. In IL‐22‐deficient host mice, Reg3β and Reg3γ mRNA was dramatically reduced which was associated with increased epithelial damage and loss of intestinal stem cells 9. Thus, IL‐22 appears as a crucial host‐derived factor modulating susceptibility to GVHD (Figure 2). The known cellular sources of IL‐22 are T cells and ILC3 and very recently an IL‐22 reporter mouse revealed that the major and most stable IL‐22‐producing cells are the NKp46^−^ CD4^−^ ILC3, while only a minority of NKp46^+^ ILC3 expressed IL‐22 35. In line with these recent findings, only IL‐22 produced by the IL‐23‐responsive RORγt^+^ CCR6^+^ NKp46^−^ ILC3 proved to be essential in the protection against GVHD 9. Importantly, intestinal IL‐22 producing ILC3 were of recipient origin, as being radio‐resistant they persisted following lethal conditioning radiotherapy, bone marrow transplantation, and even after donor T cell reconstitution in the lamina propria.

The significance of ILC3‐derived IL‐22 after immunodepletion was also shown in the enhanced regeneration of thymic epithelial cells and in the reconstitution of thymic T cell compartments 36. The identified cellular cascade after thymic injury consisted of upregulation of radio‐resistant CD103^+^ DCs providing IL‐23, which in turn stimulated IL‐22 release from RORγt^+^ CCR6^+^ NKp46^−^ LTi cells in the thymus. This supported proliferation and recovery of the epithelial microenvironment and boosted thymopoiesis.

### Lessons from human studies

Recently, human peripheral blood ILC recovery was followed longitudinally in acute myeloid leukemia (AML) patients after induction chemotherapy and allogeneic HCT 10. Human ILC were depleted after induction and conditioning chemotherapy and their complete reconstitution post‐HCT required more than 6 months. Reconstituted ILC were donor‐derived and all ILC subsets showed higher expression of the activation marker CD69 and the proliferation marker Ki‐67 as compared to ILC from healthy individuals' blood. Already after induction chemotherapy, but prior to allogeneic HCT, ILC2 and ILC3 subsets showed a dichotomy in CD69 expression. Patients with increased expression of gut‐homing markers on CD69^high^ ILC showed lower incidence of intestinal GHVD. Additionally, NCR^+^ ILC3 appeared rapidly in the peripheral blood after HCT, while this ILC subset is absent in the circulation under homeostatic conditions. Notably, 12 weeks after HCT, the circulating NCR^+^ ILC3 count was highest in patients that did not develop GVHD. Further associations were observed between the phenotype of reconstituted ILC and the development of aGVHD; (1) the activated CD69^high^ ILC subsets expressed more homing markers for gut (α4β7, CCR6) and skin (CLA, CCR10), (2) the appearance of CD69^high^ ILC, especially NCR^+^ ILC3, lowered the incidence of aGVHD after HCT (Figure 2). In contrast, patients with a more naïve ILC phenotype, lacking expression of gut‐ and skin‐homing markers, more frequently developed intestinal aGVHD 10.

These findings point toward an important role of IL‐22 producing ILC3 in preventing GVHD development in humans and are in line with previous observations in a mouse model of acute intestinal GVHD 9, 10. It is very likely that IL‐22 producing NCR^+^ ILC3, as epithelial tissue protecting cells, are crucial in the reintegration of mucosal barriers after immune depletion and allogeneic HCT. Thereby, NCR^+^ ILC3 suppress further tissue damage, alloreactive T cell activation and the occurrence of GVHD (Figure 2) 31. Furthermore, IL‐22 cytokine‐based therapy has already been considered for prevention of GVHD and lung transplantation complications 37. The active status and gut/skin homing receptor expression of ILC, especially the IL‐22‐producing ILC3, seems to minimize GVHD development following HCT. Therefore, screening patients for activation molecules and homing integrins on ILC3 prior to allogeneic HCT can be critical in anticipating the patient's sensitivity to subsequent GVHD. In case that this association between ILC3 and susceptibility to GVHD is further confirmed in future independent cohorts, supplying acute myeloid leukemia patients with IL‐22 or IL‐22‐producing ILC3 might be a mean to prevent GVHD. IL‐22 could be a relatively safe target in enhancing mucosal healing, since it does not directly affect immune cell functions. However, possible adverse effects might occur, for example, due to dysregulated responses of keratinocytes in the skin 20, 37. Of note, in an earlier large‐scale clinical study investigating human gastrointestinal GVHD risk factors, Reg3α protein was identified as indicator of higher incidence for developing GVHD after allo‐HCT 38. As mentioned previously, the antimicrobial Reg3 proteins are downstream of IL‐22/IL‐22R pathway, mainly produced by epithelial cells and ISC. This finding suggests possible differences in the underlying mechanism of GVHD development in mouse and human.

## Possible Roles for Other ILC Subsets in GVHD

In the above mentioned study, in the patients that did not develop GVHD, not only the NCR^+^ ILC3, but all circulating ILC subsets expressed high levels of the activation marker CD69 and either skin‐ or gut‐homing integrin molecules 10. This suggests that ILC1, ILC2, and the NCR^−^ ILC3 might also be associated with protection against GVHD. Particularly, the CD69^high^ ILC2 and NCR^−^ ILC3 subsets expressed significantly higher levels of the gut‐homing marker α4β7 in patients without GVHD. However, the authors stated that further larger cohorts are necessary for studying the relation between these ILC subsets and GVHD sensitivity. Theoretically, these ILC subsets and their released cytokines and other mediators might also contribute to the GVHD pathology.

## ILC2 in GVHD

Mouse studies based on Th2 cells provided evidence for a protective role for type 2 cytokines, such as IL‐4, IL‐5, IL‐9, and IL‐13, in GVHD 31. These cytokines are also extensively produced by ILC2. All together, variable effects had been reported about Th2 cytokines. Interestingly, at least in mice, ILC2 produce the EGF‐related factor amphiregulin, which was shown to trigger epithelial tissue repair and restore lung tissue homeostasis after influenza virus infection 2. This notion suggests that ILC2 could possibly affect the sensitivity to GVHD via boosting epithelial cell regeneration after the conditioning therapy‐induced tissue damage. However, the experimental data that support such a role of ILC2 in GVHD development are still missing.

## NCR^−^ ILC3 in GVHD

NCR^−^ ILC3 and NCR^+^ ILC3 produce IL‐17. In mouse models of GVHD, IL‐17 produced by Th17 cells turned out to be sufficient but not necessary to induce GVHD 31. Notably, IL‐17 contributed to the early development of CD4^+^ T cell‐mediated GVHD by triggering pro‐inflammatory cytokines. Elsewhere, adoptive transfer of Th17 cells resulted in lethal GVHD, which was dependent on TNF‐α and IL‐17 production. In contrast, selective depletion of Th17 cells by targeting the Th17‐specific RORγt transcription factor did not affect the GVHD development in a mouse model. There is no direct evidence for a role of NCR^−^ ILC3‐derived IL‐17, but based on the reports about Th17 cells, IL‐17 might not be crucially involved in the GHVD pathology.

## Conclusions and Perspectives

Despite advances in our understanding and management of allogeneic hematopoietic cell transplantation immunology field, GVHD remains the major challenge and limitation of allogeneic HCT therapy. Besides mainly focusing on the donor immune system, the role of host defense responses is becoming increasingly recognized. Recent reports described the central importance of recipient sensitivity to developing acute intestinal GVHD that is dictated by the homeostatic status of the gut epithelial tissue. Conditioning chemo‐ and radiotherapy causes damage to the intestinal epithelial crypts and the epithelial progenitor cells, which are responsible for regenerating the entire gut mucosal lining. Group 3 ILC have recently been identified as sentinels of the intestinal epithelial crypts via production of IL‐22, a cytokine that is crucial in maintaining epithelial cell integrity. It has turned out that the appearance of activated human ILC3 in the circulation positively correlates with enhanced protection against GVHD development after HCT. There is a strong reason to believe that the IL‐22 producing ILC3 are able to diminish the risk for GVHD by triggering the regeneration of the intestinal mucosa. Future research should dissect the exact contribution of ILC3 and possibly also other ILC subsets in GVHD immunology. Additionally, larger clinical studies are required for confirming the critical importance of ILC in regulating GVHD susceptibility.

## Outstanding Questions

1. What regulates the appearance of NCR^+^ ILC3 in the blood circulation after conditioning chemotherapy?
2. Besides IL‐22 which other NCR^+^ ILC3‐derived factors might be important in the prevention against GHVD after allo‐HCT?
3. Are ILC1 and ILC2 involved in the development of GVHD?

## Disclosure

The authors of this manuscript have no conflicts of interest to disclose as described by the *American Journal of Transplantation*.
